# Uterine Preservation Following Catastrophic Hemorrhage From an Undiagnosed First-Trimester Placenta Increta: A Case Report and Review of the Literature

**DOI:** 10.7759/cureus.104233

**Published:** 2026-02-25

**Authors:** Khalid M Akkour, Ahmed Sherif Abdel Wahab, Ghadeer Ahmad Alneel, Mazin Baazeem

**Affiliations:** 1 Obstetrics and Gynecology, Faculty of Medicine, King Saud University, Riyadh, SAU; 2 Obstetrics and Gynecology, Dr. Sulaiman Al Habib Women’s Health Hospital, Riyadh, SAU; 3 Obstetrics and Gynecology, Consultant, Riyadh, SAU; 4 Obstetrics and Gynecology, Ain Shams University, Cairo, EGY; 5 Obstetrics and Gynecology, Dr. Sulaiman Al Habib Medical Group, Riyadh, SAU; 6 Gynecologic Oncology, King Fahd Medical City, Riyadh Second Health Cluster, Riyadh, SAU

**Keywords:** internal iliac artery ligation, missed abortion, placenta accreta spectrum, placenta increta, placental excision

## Abstract

Placenta accreta spectrum (PAS) in the first trimester is a rare but potentially fatal condition. Preoperative diagnosis during early pregnancy remains extremely challenging, particularly in individuals with a previous cesarean delivery. We describe the case of a 35-year-old woman (gravida 3, para 2) with two prior cesarean deliveries who presented at eight weeks of gestation for the management of a missed abortion. Despite her high-risk status, PAS was not suspected on initial ultrasound. Medical induction with misoprostol resulted in sudden massive hemorrhage and hemorrhagic shock 12 hours later. An emergency dilation and curettage (D&C) was unsuccessful in controlling the bleeding. The patient was stabilized with immediate exploratory laparotomy, which revealed a 4 × 4 cm placenta increta. Hemostasis was achieved through bilateral internal iliac and uterine artery ligation, excision of the placental mass, and repair of the uterine defect, allowing for uterine preservation and a smooth postoperative course. This report underscores the significant risk of catastrophic hemorrhage from previously unrecognized PAS in patients with prior cesarean deliveries and highlights a critical diagnostic gap in early pregnancy care. It demonstrates that while fertility-sparing surgery is achievable with rapid intervention, greater emphasis should be placed on prevention through enhanced preoperative risk assessment and optimized imaging protocols for high-risk patients.

## Introduction

Placenta accreta spectrum (PAS) is a life-threatening obstetric condition characterized by severe hemorrhage, massive transfusion, emergency hysterectomy, and multi-organ damage. Its incidence has risen dramatically in recent decades, largely due to the increasing rate of cesarean deliveries, increasing from one in 4000 births in the 1970s to approximately one in 500 today. While it is most commonly diagnosed in the second or third trimester, PAS is exceptionally rare and diagnostically challenging in the first trimester, with fewer than 100 cases described in systematic reviews, highlighting its rarity at this early gestational age [1].

The early and accurate diagnosis of CSP, particularly when accompanied by placenta increta, remains a formidable clinical challenge in the first trimester. While transvaginal sonography (TVS) with color Doppler serves as the primary first-line screening tool, its limitations in delineating subtle microvascular perfusion patterns can lead to misdiagnosis as a cervical pregnancy or inevitable abortion. Contrast-enhanced ultrasound (CEUS) has emerged as a highly promising modality, providing dynamic, real-time visualization of placental blood flow and the depth of myometrial invasion. This technique provides a superior, more accessible evaluation for morbidly adherent placenta compared with MRI in urgent scenarios, enabling a more confident and timely diagnosis [2-4]. We present a case involving a patient with prior cesarean deliveries who suffered catastrophic hemorrhage during evacuation of a missed abortion, ultimately revealing a life-threatening first-trimester placenta increta.

## Case presentation

Patient information

The patient was a 35-year-old woman, gravida 3, para 2, with a history of two prior cesarean deliveries. She presented for management of a missed abortion diagnosed at approximately eight weeks of gestation and confirmed by ultrasound. A transvaginal ultrasound performed by a staff obstetrician confirmed a nonviable intrauterine pregnancy at eight weeks, consistent with a missed abortion. The gestational sac was noted to be located in the lower uterine segment. Its relationship to the previous cesarean scar was not specified in the report, and targeted Doppler assessment of placental perfusion was not undertaken. The official report concluded that there was no evidence of abnormal placentation. Her obstetric history was notable for two prior cesarean sections, which increased her risk of abnormal placentation, particularly placenta increta, although this possibility was not considered at the time of the initial diagnosis. Although her previous cesarean deliveries were uncomplicated, they predisposed her to potential placental implantation abnormalities in the current pregnancy.

**Figure 1:**
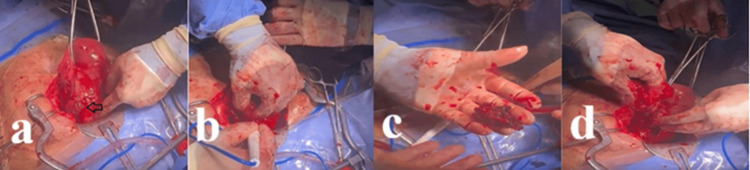
Intraoperative findings during exploratory laparotomy for massive hemorrhage (a) The anterior uterus is exposed, revealing a 4x4 cm hemorrhagic bulge (black arrow) at the site of the prior cesarean scar on the lower uterine segment. (b) Sharp dissection is used to separate and remove the adherent placental tissue (placenta increta). (c) The surgically excised conceptus and placental tissue. (d) The resulting uterine defect after excision, before multilayer repair. Hemostasis was achieved before this via bilateral internal iliac and uterine artery ligation

The primary therapeutic approach shifted urgently from medical management (Table 1), initiated with an 800 mcg dose of vaginal misoprostol for induction, to surgical intervention due to severe hemorrhage. An emergency dilation and curettage (D&C) was initiated but was promptly converted to laparotomy because of suspected uterine perforation. An immediate exploratory laparotomy was performed by a team led by a gynecologic oncologist with expertise in pelvic surgery (Figure 1). Upon entering the abdomen, a 4 × 4 cm hemorrhagic bulge was identified on the anterior lower uterine segment at the site of the previous cesarean scar. To control the life-threatening bleeding, bilateral ligation of the anterior divisions of the internal iliac arteries was performed, followed by ligation of the uterine arteries, resulting in a marked reduction of pelvic arterial inflow. The abnormal placental tissue was then sharply excised, and the resulting uterine defect was repaired in layers.

**Table 1:** Case presentation in chronological pattern

Timing of the event	Event/intervention	Details and outcome
The day before surgery	Initial presentation and diagnosis	Patient para 2 CS presents with a history of pregnancy confirmed and spotting. Transvaginal ultrasound confirms a missed abortion at eight weeks. No abnormal placentation is reported. Her lab values were normal with Hb 11 gm/dl
Morning of surgery	Medical management initiated	A trial of medical induction begins with the administration of vaginal misoprostol 800 mcg per FIGO protocol for first-trimester management. Per protocol, subsequent doses of 400 mcg can be repeated every three hours if needed. The patient is admitted for inpatient monitoring due to her history of two prior cesarean sections, a significant risk factor for complications
Evening of surgery	Acute complication and emergency surgery	The patient develops a sudden, severe vaginal hemorrhage, manifesting as hypotension (BP: 90/60 mmHg) and tachycardia (HR: 100 bpm), signaling the onset of hypovolemic shock. An emergency dilation and curettage is initiated to control the bleeding. During the procedure, her condition deteriorates rapidly with profound hypotension (BP: 70/40 mmHg) and worsening tachycardia (HR: 120 bpm) due to torrential, uncontrollable hemorrhage. A uterine perforation is suspected. Concurrently, emergency blood products are prepared and transfused after stat labs are drawn, which revealed an acute drop in hemoglobin to 9 gm/dL
During laparotomy	Surgical escalation, definitive diagnosis, and management	Due to the life-threatening hemorrhage, the procedure is escalated to an immediate exploratory laparotomy
		A blood transfusion is initiated, with the patient receiving two units of packed red blood cells to correct anemia and stabilize hemodynamic status. Laparotomy reveals the definitive diagnosis: a 4x4 cm placental bulge, confirming placenta increta. Bilateral uterine artery ligation and bilateral internal iliac artery ligation are performed to control the catastrophic bleeding. The abnormal placental tissue is excised, and the uterine defect is repaired
Postoperative	Recovery and discharge	The patient's condition stabilizes. She has an uneventful recovery and is discharged with a plan for follow-up monitoring

The patient received two units of packed red blood cells and achieved hemodynamic stability postoperatively, with hemoglobin improving to 10.5 g/dL (Table 2). Postoperative follow-up included serial monitoring of beta-hCG levels until they returned to zero.

**Table 2:** Serial laboratory investigations for the patient

Laboratory parameter	At presentation	During hemorrhage	Post-transfusion/stabilization	Reference range
Hemoglobin (Hb)	11.0 g/dL	9.0 g/dL	10.5 g/dL	12.0 - 15.5 g/dL
Hematocrit (Hct)	33%	27%	32%	36% - 48%

## Discussion

This case highlights the significant dangers and management challenges associated with unrecognized first-trimester PAS in a high-risk patient. Despite the patient’s history of two prior cesarean sections, PAS was not identified preoperatively, underscoring an important lesson regarding diagnostic error. The statement that “no abnormal placentation was identified” reflects a complex diagnostic breakdown rather than a simple oversight. First, the limitations of standard ultrasonography likely played a central role. The initial scan, performed without the use of advanced Doppler imaging or contrast-enhanced techniques, may have failed to detect key early signs of PAS, such as a low-lying gestational sac, multiple placental lacunae, or thinning or absence of the myometrial layer at the prior cesarean scar site. This case underscores the persistent gap between guideline recommendations for high-risk patients and their implementation in routine clinical practice.

Second, cognitive biases likely influenced the diagnostic process. The identification of a “missed abortion” may have narrowed the clinical focus to miscarriage management, thereby limiting consideration of alternative or coexisting pathology. The framing effect may also have contributed, as the patient was primarily categorized under the diagnosis of miscarriage rather than being approached as a high-risk candidate for PAS based on her cesarean history. A different clinical framing might have prompted a more comprehensive evaluation, potentially including expert ultrasound review.

Our case is not an anomaly but rather reflects the predominant clinical pattern of first-trimester PAS described in the systematic review by Guzmán López et al. [1]. The most concerning aspect is the representative nature of the diagnostic failure. Like 85 percent of patients in that review, our patient had a prior cesarean delivery; like 74 percent, she presented with miscarriage; and like 79 percent, PAS was not suspected preoperatively. These parallels underscore that failure to recognize PAS in early pregnancy remains a widespread systems-level problem rather than an isolated lapse in clinical judgment. In contrast, the atypical element in our case was the outcome, specifically successful uterine preservation. Whereas hysterectomy was the most common outcome reported in the review, our experience demonstrates that this result is not unavoidable and highlights the potential role of timely salvage surgical techniques.

The catastrophic emergency we encountered was potentially preventable. This contrasts sharply with the case reported by Mai et al. [4], which illustrates a model of anticipatory and protocol-driven management. In that report, a similarly high-risk patient (G3P2 with two prior cesarean deliveries) presented with bleeding, but persistently elevated β-hCG levels prompted repeat imaging. Critically, the use of CEUS enabled dynamic, real-time assessment of placental hypervascularity and depth of myometrial invasion, confirming placenta increta before surgery. This facilitated a planned, minimally invasive laparoscopic excision and avoided catastrophic hemorrhage. The key lesson extends beyond differing outcomes and instead underscores the need for system-level change in clinical practice. Implementing a protocol that incorporates advanced imaging, such as CEUS or MRI, for high-risk patients with atypical presentations could reduce the need for emergency salvage surgery and should be strongly considered as an emerging standard of care.

Furthermore, a high index of suspicion for PAS must extend beyond the classic high-risk profile. The report by De Gennaro et al. [5] serves as a critical reminder that PAS can occur even in patients without prior uterine surgery. In that case, the presentation mimicked a molar pregnancy, and unrecognized placental invasion led to curettage that precipitated catastrophic hemorrhage requiring emergency hysterectomy. Therefore, although prior cesarean delivery remains the strongest risk factor, PAS should be included in the differential diagnosis of unexplained first-trimester bleeding or abnormal β-hCG trends in all patients, regardless of surgical history. This case reinforces that the precipitating event is often an uninformed surgical evacuation, independent of obstetric background.

Our favorable outcome was not inevitable but rather the result of prompt escalation of care and immediate access to advanced surgical expertise. The decision to proceed directly to laparotomy and to perform bilateral internal iliac artery ligation proved to be a life-saving intervention that established the hemodynamic conditions necessary for uterine preservation. Although technically successful, this achievement does not offset the initial diagnostic shortcoming; instead, it highlights the substantial resources and specialized skills required to manage a complication that should ideally be anticipated and prevented. It underscores the need for centers managing high-risk pregnancies to maintain not only operative preparedness but, more importantly, structured and rigorous preoperative diagnostic pathways.

Strengths and limitations

A key strength in the management of this case was the prompt identification of hemorrhagic shock and the decisive transition to emergency laparotomy, which permitted direct visualization and control of the bleeding source. Bilateral internal iliac artery ligation proved to be a pivotal, life-saving intervention that significantly reduced pelvic arterial inflow and established a stable operative field, thereby enabling meticulous excision of the placenta increta and layered uterine repair. The principal limitation of this case was the failure to consider PAS in the preoperative differential diagnosis, resulting in a preventable life-threatening emergency. This shortcoming extended beyond the technical limitations of standard ultrasonography and reflected both cognitive bias and systems-level deficiencies in risk stratification and imaging protocols. Management diverged from the expected standard of care for a high-risk patient, which should have included heightened clinical suspicion, consideration of advanced imaging modalities, and expert sonographic evaluation. Ultimately, this case highlights the consequences of suboptimal preoperative assessment while also demonstrating the effectiveness of rapid, well-coordinated rescue management.

## Conclusions

This report offers two critical lessons for the management of early pregnancy in high-risk patients. First, it serves as a stark warning about the potentially devastating consequences of diagnostic failure. Despite well-established risk factors, our patient underwent uterine evacuation without suspicion of placenta accreta, precipitating a predictable and nearly fatal hemorrhage. Second, it demonstrates that with rapid escalation of care, advanced surgical expertise, and techniques such as internal iliac artery ligation, hysterectomy can be avoided even in extreme emergencies. The central message is that reliance on standard ultrasonography and cognitive anchoring to common diagnoses such as miscarriage is inadequate. Preventing similar events requires an evolution in clinical practice toward a higher standard of care, including routine implementation of advanced imaging protocols and structured multidisciplinary planning for any patient with a prior cesarean delivery undergoing pregnancy termination or miscarriage management. Ultimately, the objective should not be merely to improve crisis management, but to establish systems that render such catastrophes increasingly uncommon.
